# *Metasequoia glyptostroboides* potentiates anticancer effect against cervical cancer via intrinsic apoptosis pathway

**DOI:** 10.1038/s41598-020-79573-8

**Published:** 2021-01-13

**Authors:** Hoomin Lee, Cheolwoo Oh, Suji Kim, Debasish Kumar Dey, Hyung Kyo Kim, Vivek K. Bajpai, Young-Kyu Han, Yun Suk Huh

**Affiliations:** 1 Department of Biological Engineering, NanoBio High-Tech Materials Research Center, Inha University, 100 Inha-ro, Nam-gu, Incheon, 22212 Republic of Korea; 2 Department of Biotechnology, Daegu University, Gyeongsan, 38453 Republic of Korea; 3 Department of Biomaterials Research Center, GENPEAU Corporation, Incheon, 21990 Republic of Korea; 4 Department of Energy and Materials Engineering, Dongguk University-Seoul, 30 Pildong-ro 1-gil, Seoul, 04620 Republic of Korea

**Keywords:** Cancer prevention, Cancer therapy

## Abstract

This study was undertaken to investigate the anticancer effects of organic extracts derived from the floral cones of *Metasequoia glyptostroboides*. Dried powder of *M. glyptostroboides* floral cones was subjected to methanol extraction, and the resulting extract was further partitioned by liquid–liquid extraction using the organic solvents *n*-hexane, dichloromethane (DME), chloroform, and ethyl acetate in addition to deionized water. HeLa cervical and COS-7 cells were used as a cancer cell model and normal cell control, respectively. The anticancer effect was evaluated by using the Cell Counting Kit-8 assay. The viability of COS-7 cells was found to be 12-fold higher than that of the HeLa cells under the administration of 50 µg/ml of the DME extract. Further, the sub-G1 population was determined by FACS analysis. The number of cells at the sub-G1 phase, which indicates apoptotic cells, was increased approximately fourfold upon treatment with the DME and CE extracts compared with that in the negative control. Furthermore, RT-qPCR and western blotting were used to quantitate the relative RNA and protein levels of the cell death pathway components, respectively. Our results suggest that the extracts of *M. glyptostroboides* floral cones, especially the DME extract, which possesses several anticancer components, as determined by GC–MS analysis, could a potential natural anticancer agent.

## Introduction

Cervical cancer affects many women in developing countries and has a high mortality rate. Although the cervical cancer screening program has reduced the incidence and mortality of cervical cancer, the incidence among young women remains a public health problem^1^. In addition, the administration of chemotherapeutic agents and multi-drug resistance are accompanied by severe side effects that cause negative gynecological and obstetric outcomes^2,3^.

Tumor cells in the ovary can metastasize to the lymphatic and circulatory systems if not blocked by the anatomical barriers^4^. Surgical resection and systemic chemotherapy can affect drug efficacy. Consequently, high chemotherapeutic doses that are adversary to human health are the only options to treat the mucosal and epithelial membranes of the affected tissues. Doxorubicin (DOX) is an efficient chemotherapeutic agent that is widely used for the treatment of various cancers^5^. However, adversary side effects, poor bio-distribution, and high toxicity to non-cancerous cells limit the use of DOX^6,7^.

Targeting apoptosis avoidance, a major feature of cancers, is the most effective nonsurgical strategy for the treatment of cancers because it is not specific to the cause or type of cancer^8^. Apoptosis is mediated by an intrinsic or extrinsic pathway based on the origin of the apoptotic stimulus. These pathways are also known as mitochondrial and death receptor pathways, respectively^8^. The intrinsic apoptosis pathway is activated inside the cell by the Bcl-2 protein family and is independent of receptor signal transduction^8,9^. The extrinsic pathway is primarily mediated by the signaling through membrane-bound receptors belonging to the tumor necrosis factor (TNF) superfamily called the “death domain”^9^.

*Metasequoia glyptostroboides* Miki ex Hu (*M. glyptostroboides*) is a deciduous conifer from the red-wood family of Cupressaceae and is distributed in Europe as well as many parts of East Asia and North America^10^. Herbal plant-based extracts have shown enormous potential in the treatment of various diseases, including cervical cancer. Although *M*. *glyptostroboides-*derived extracts or secondary metabolites have been found to exhibit numerous biological and pharmacological activities^11–13^, the effect of *M. glyptostroboides* floral cone extracts on cervical cancer has not been addressed to date.

Therefore, in this study, *M. glyptostroboides* floral cone extracts prepared with various organic solvents were assessed for their anticancer effects on the cervical cancer cell line HeLa cells versus their normal counterparts, COS-7 cells. Also, chemical component analysis of the DME extract was performed using GC–MS analysis.

## Methods

### Materials

The organic solvents *n*-hexane, dichloromethane, chloroform, and ethyl acetate were purchased from Daejung (Korea). Dulbecco’s Modified Eagle’s Medium (DMEM), penicillin/streptomycin, fetal bovine serum (FBS), phosphate-buffered saline (PBS), and trypsin were purchased from Gibco (Carlsbad, CA). The Cell Counting Kit-8 (CCK-8) was purchased from Dojindo Co. Ltd. (Beijing, China).

### Sample preparation

**Figure 1:**
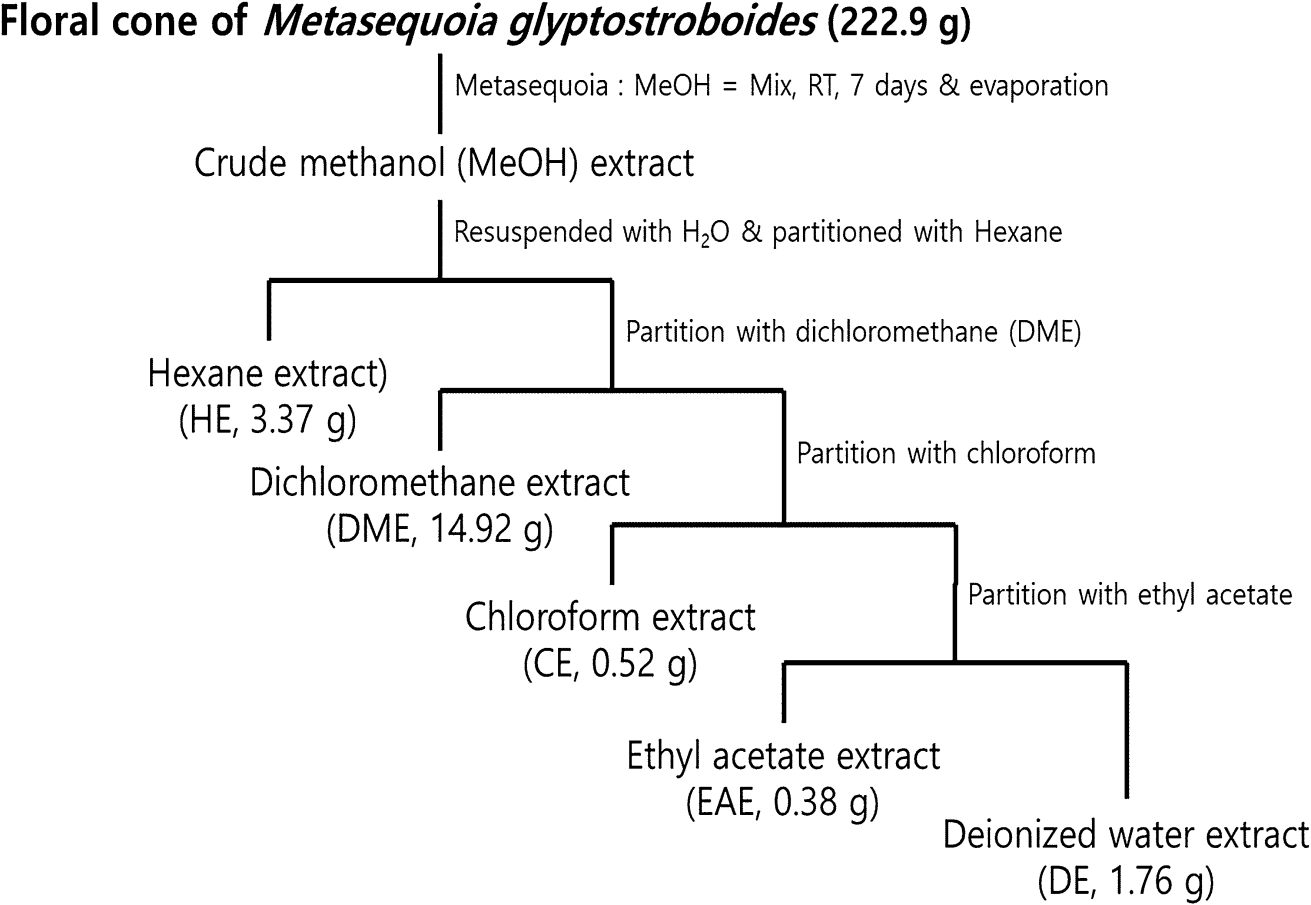
Partition of different solvent extracts through liquid–liquid extraction.

The floral cone powder *of M. glyptostroboides* was subjected to methanol extraction at a ratio of 1:10 (w/v) for 1 week and then filtered through a 0.45-μm Whatman No. 1 filter paper. The supernatant was dried using a rotary evaporator (N-1110S-W, Eyela, Tokyo, Japan), resulting in the crude methanol extract (ME) of *M. glyptostroboides* floral cones with a yield of 9.40%. To prepare different organic extracts, ME was further dissolved in deionized (DI) water and successively partitioned using *n*-hexane (HE), dichloromethane (DME), chloroform (CE), and ethyl acetate (EAE) solvents. Each solvent layer was separated and dried using a rotary evaporator, resulting in the crude HE, DME, CE, and EAE extracts of *M. glyptostroboides* floral cones. To prepare the test samples, each extract sample was dissolved in dimethyl sulfoxide (DMSO). A detailed extraction procedure is provided in Fig. 1.

### Cell cultures

HeLa (Human cervical carcinoma cell) and COS7 (African green monkey kidney cell) cells were cultured in DMEM medium with 10% FBS and 1% penicillin/streptomycin. Cells were incubated at 37 ℃ in humidified air with 5% CO_2_.

### Gas chromatography-mass spectroscopy (GC–MS) analysis

GC–MS anlysis of DME extract was performed using a GC-Thermo Jeol JMS700 appratus following our previously reported method, and componentes were identified using GC–MS-based NIST and Willey library^14^.

### Cytotoxicity assay

For cytotoxicity assay, COS7 and HeLa cells were incubated with various concentrations of HE, DME, CE, EAE, and DE extracts of *M. glyptostroboides* floral cones. After 24 h incubation, cells were washed with PBS three times. Then, the cell viability was measured using a CCK-8 cell counting kit following the manufacturer’s protocol.

### Nuclear fragmentation analysis

Hoechst staining^15^ was performed to examine the nuclear fragmentation effect of HE, DME, CE, EAE, and DE extracts of *M. glyptostroboides*. The HeLa cells were grown in 12-well-pate and treated with 50 µg/ml of HE, DME, CE, EAE, and DE extracts for 24 h. Finally, cells were treated with 1 µg/ml of Hoechst 33342 stain. After 15 min staining cells were washed with PBS (pH 8) and visualized under EPI fluorescence microscope (Nikon, Japan) and apoptotoc index was calculated as the percentgae of apoptotic nuclei compared to the total number of cells^15^.

### Quantification of sub-G1 phase by flow cytometry analysis (FACS)

FACS analysis was carried out to determine the cytotoxic effecet of HE, DME, CE, EAE, and DE extracts of *M. glyptostroboides*. The sub-G1 population was quantified using propidium iodide (PI; Sigma-Aldrich) staining and a cellometer. HeLa cells were seeded in 6-well plates at a density of 1 × 10^6^ cells per well, cultured for 24 h and then treated with 50 μg/ml of the HE, DME, CE, EAE, or DE extract for 24 h. Cells were harvested with trypsinization and fixed with 80% cold ethanol in PBS for 30 min. Cells were then washed twice with cold PBS and centrifuged at 2000 rpm. The pellet was resuspended in PBS and stained with 50 μg/ml PI in PBS containing 100 μg/ml RNase A. Cells were then incubated at 37 ℃ for 40 min. Afterward, their DNA contents were analyzed using the cellometer instrument.

### Western blot analysis

HeLa cells were pretreated with 50 µg/ml of the HE, DME, CE, EAE, or DE extract of *M. glyptostroboides* floral cones. Total protein was obtained by lysing the cells with RIPA buffer containing protease and phosphatase inhibitors (ThermoFisher Scientific, UK). Protein samples were subjected to SDS-PAGE and electro-transferred onto polyvinylidene difluoride membranes (Millipore, Burlington, MA). The membranes were blocked using 5% skim milk in Tris-buffered saline-Tween 20 (TBST). The membranes were incubated overnight at 4 ℃ with the primary antibodies and then washed with TBST. The membranes were subsequently treated with the appropriate secondary antibodies for 4 h. β-actin was used as an internal standard. Image J software was used to analyze the band intensities.

### RT-qPCR analysis

The mRNA levels of apoptosis markers in HeLa cells were assessed using RT-qPCR. Total RNA was collected using the TRIzol reagent (Life Technologies, Carlsbad, CA) and reverse-transcribed using the PrimeScript RT reagent Kit (Takara Bio Inc., Kusatsu, Japan). The CFX96 system (Bio-Rad Laboratories, Hercules, CA) and iQ SYBR Green Supermix (Bio-Rad Laboratories) were used for qPCR. β-actin mRNA levels were used to normalize p53, B-cell lymphoma 2 (Bcl-2), and Bcl-2-associated X (BAX) mRNA levels. The 2^(−ΔΔCT)^ method was used to determine the relative mRNA levels. The primer sequences used in this study are provided in Supplementary Table S1.

### Detection of reactive oxygen species (ROS) generation

The detection of ROS generation assay was according to previous studies with some modification^16^. To detect ROS at chemical level, a 500 µl sample of 1 mM 2′,7′-dichlorodihydrofluorescein diacetate (DCF-DA; 287810, MERCK MILLIPORE, German) was reacted with 10 mM NaOH (2 ml) for 30 min to achieve complete deacetylation in the darkroom. The mixture solution was then neutralized with 10 ml PBS. Each sample in PBS was mixed with DCF-DA solution and horseradish peroxidase (HRP; P8375, SIGMA, USA) (2.2 unit per ml) at a ratio of 1:1:1 and reacted in a darkroom for 30 min. Centrifugation proceeded at 13,000 rpm and 4 ℃ for 10 min. The supernatant solution was moved to a 96 well black plate and the fluorescence intensity of DCF was observed with excitation and emission wavelengths at 485 nm and 535 nm, respectively. The standard curve was obtained from a H_2_O_2_ solution.

Further, the generation of ROS at cellular level was evaluated using DCF-DA (Cellular ROS assay kit, Abcam). In the presence of HRP and H_2_O_2_, DCF-DA is converted to highly fluorescent 2′,7′-dichlorodihydrofluorescein (DCF). The ROS assay was performed according to the supplier's instructions. The confluent cells incubated at the concentration of 50 µg/ml per extract for 6 h in 12-well cell plates were treated with 1 mM of H_2_O_2_ for 30 min. The cells were twice washed with PBS and incubated with 10 mM DCF-DA for 40 min at 37 ℃ in the dark. The cells were then washed twice with PBS and analyzed by a microplate reader at an excitation and emission wavelength of 485 nm and 530 nm, respectively.

### Statistical analysis

The data presented as the mean ± standard deviation from three independent experiences was analyzed by student’s t-test with a p-value of < 0.01 considered as significant for the differences.

## Results and discussion

### GC–MS analysis of DME

GC–MS analysis of DME resulted in the indentification of 45 different chemical compounds (Supplementary Table S2). The majority of compounds belonged to the organic acids, terpenes and phenolic compounds, especially terpenes and quinones such as ferruginol^17^, taxodione^18^ which contributed significant amount of the total chemical composition of DME by 14.58 and 2.21%, respectively. The anticancer activities of these compounds are mediated due to the presence of hydroxyl groups^17,18^, including other biologically active components, which have been reported to be anticancerous and/or antitumorous in nature such as estradiol^19^. The resutls of GC–MS analysis of DME confirms that the reported anticancer acitivity of DME could be mediated via these bioactive components present in the DME.

### Anticancer activities of *M. glyptostroboides* extracts

**Figure 2:**
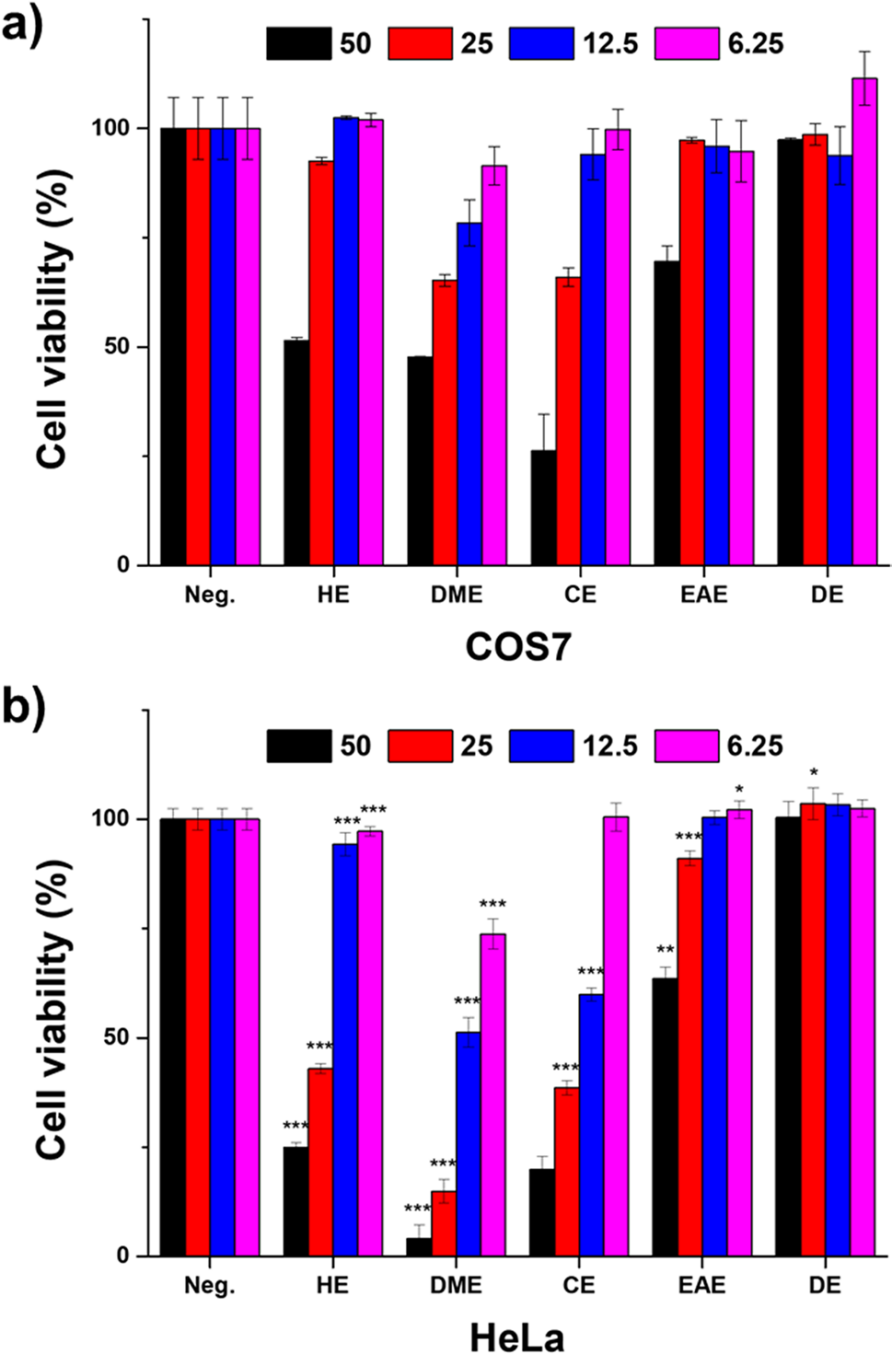
Anticancer effects of various extracts of *M. glyptostroboides.* (**a**) COS7 and (**b**) HeLa cells were incubated for 24 h with *M. glyptostroboides* extracts at 50, 25, 12.5, and 6.25 µg/ml. ***p < 0.001 indicate statistical significances compared to COS7.

The five organic extracts such as HE, DME, CE, EAE, and DE of *M. glyptostroboides* floral cones were obtained via liquid–liquid extraction of the methanol extract. In brief, dried powder of the floral cones of *M. glyptostroboides* was successively extracted with methanol and partitioned using the organic solvents *n*-hexane, dichloromethane, chloroform, and ethyl acetate in addition to DI water (Fig. 1). The cytotoxic effects of the extracts on HeLa cervical cancer cells were assessed for alongside COS7 cells as the non-cancer cell control using CCK-8. Cells were treated with the HE, DME, CE, EAE, and DE extracts for 24 h at the concentration range of 6.25, 12.5, 25, and 50 µg/ml per extract. Figure 2a,b show that the HE, DME, and CE extracts had considerable anticancer effects on HeLa cells in a dose-dependent manner. The viability of COS7 cells was 2-, 12-, and 1.3-fold higher than that of HeLa cells under the administration of 50 µg/ml of HE, DME, and CE extracts, respectively. We also confirmed low cytotoxicity of our test samples on normal cervical epithelial cells than HeLa cells (Supplementary Fig. S1). The IC_50_ values of HeLa and COS7 for HE, DE, and CE extracts were (31.89 and 52.11 µg/ml), (13.71 and 45.37 µg/ml) and (18.85 and 35.83 µg/ml), respectively, showing stronger toxicity to HeLa cells (Fig. 2). On the other hand, EAE and DE extracts did not show any difference in the cytotoxicity levels of both the tested cells lines. Previous studies have also shown the cytotoxic potentials of various plant extracts in several cancer lines including HeLa cells^20,21^.

### Quantification of sub G1 phase and nuclear disruption in HeLa cells

**Figure 3:**
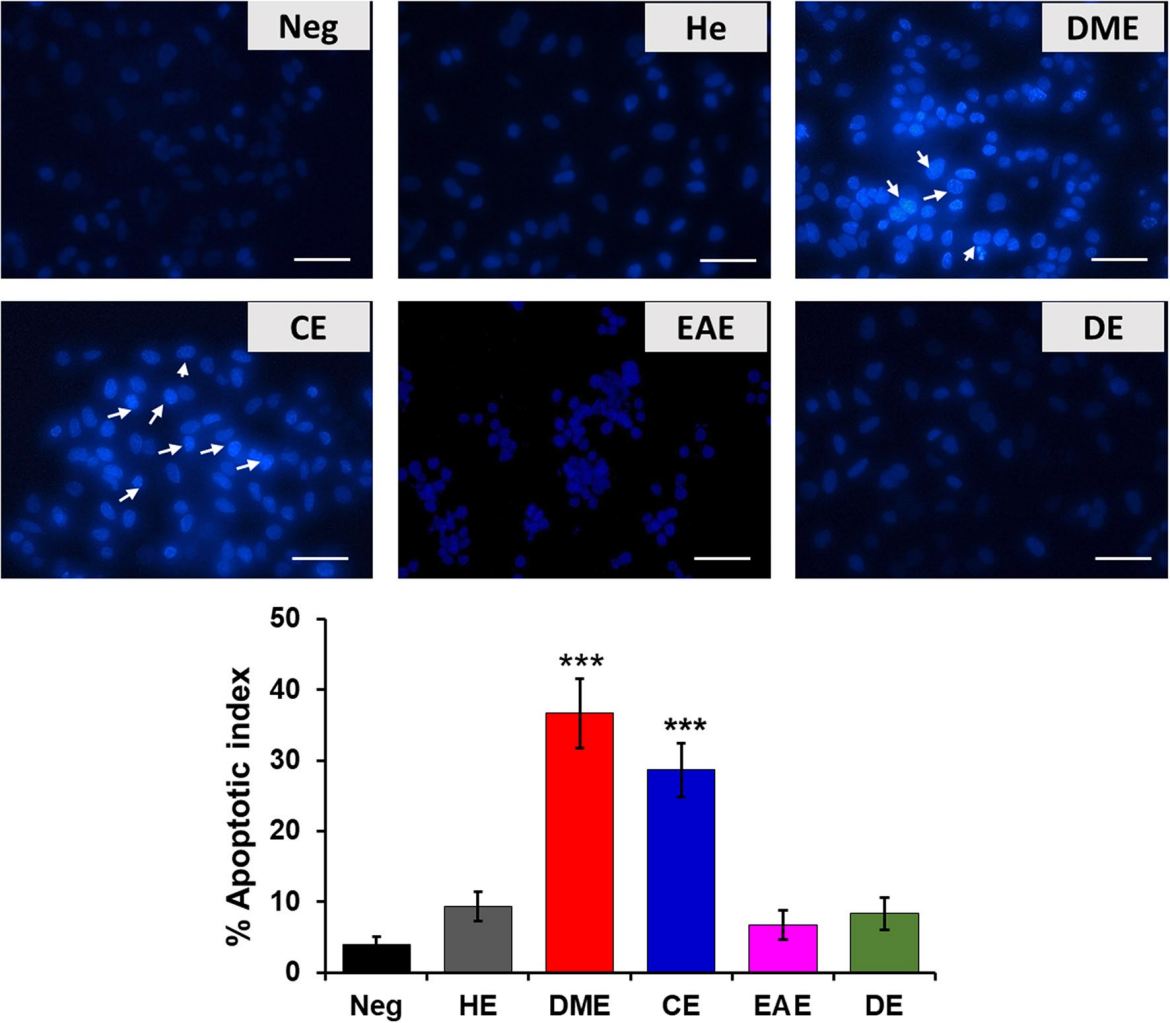
Effects of *M. glyptostroboides* extracts on the nuclei of HeLa cells. HeLa cells were incubated with *M. glyptostroboides* extracts of 50 µg/ml for 24 h. Apoptotic cells indicated as bright colored condensed and fragmented nuclei stained with Hoechst 33342 (marked by arrow).

To assess the anticancer effects of various organic extracts of *M. glyptostroboides* on HeLa cells, we performed Hoechst *33342* staining and cell cycle assay. Figure 3 shows the results of the Hoechst *33342* staining of HeLa cells treated with *M. glyptostroboides*-derived extracts. The extracts DME and CE displayed a brightly colored condensed fragmented nuclei indicated nuclear disintegration and suggesting the apoptotic cell death^22^. In contrast to this, the extracts HE, EAE, and DE did not show the presence of apoptotic nuclei. Further, staining results were quantified as apoptotic index, suggesting 36.67 ± 4.93% and 28.66 ± 3.79% apoptotic nuclei in the cells treated with DME and CE, respectively. Moreover, the phenotypic characteristic of irregular cell morphologies and membrane blebbing especially in the DME and CE extracts treated cells indicated the apoptotic morphology (Fig. 3). These results are consistent with the results of the cell viability assay shown in Fig. 1, suggesting that the extracts induced apoptosis in HeLa cells.

**Figure 4:**
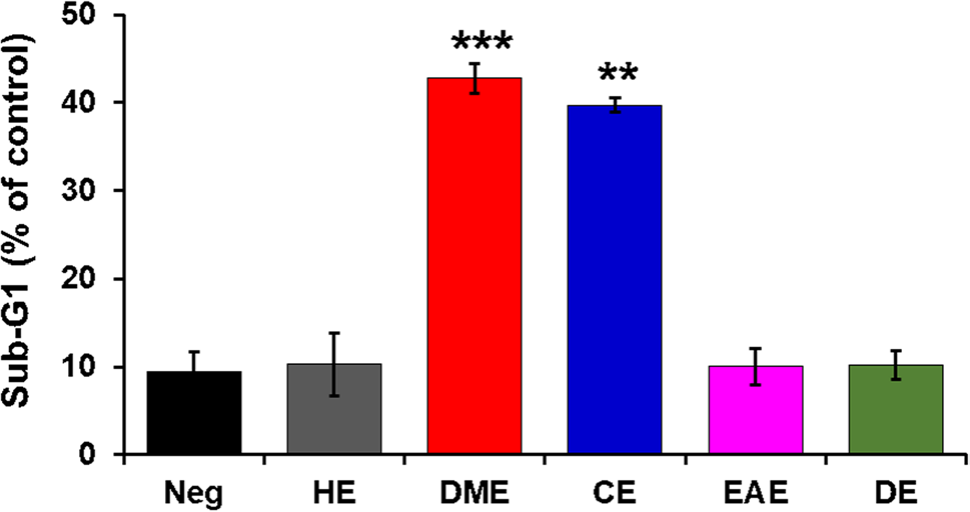
Apoptotic effect of *M. glyptostroboides* extracts examined by FACS. HeLa cells incubated with *M. glyptostroboides* extracts of 50 µg/ml for 24 h and apoptosis was analyzed as the sub-G1 fraction by FACS. **p < 0.01 and ***p < 0.001 indicates statistical significances compared to negative control.

To assess the anticancer effects of various organic extracts of *M. glyptostroboides* on HeLa cells, sub-G1 phase of cell cycle was determined by using PI-staining (Fig. 4 and Supplementary Fig. S2). PI can permeate through the damaged membranes of apoptotic cells and stains the nuclei^23,24^. Apoptotic cells accumulate in the sub-G1 phase. The proportion of the cells in the sub-G1 phase upon treatment with the DE extract was 10.2 ± 1.6% and similar to that in the negative control (9.4 ± 2.3%), HE (10.3 ± 3.6%), and EAE (10.03 ± 2.1%). The maximum sub-G1 population was observed in DME (42.76 ± 1.7%) followed by CE (39.7 ± 0.81%), which was significantly 4.5- and 4.2-folds higher compared with the negative control (Fig. 4). Collectively, the results of the cell cycle analysis, cell viability assay, (Fig. 2b), and Hoechst 33342 staining showed that the DME and CE extracts induced apoptosis, suggesting the anticancer behavior of these extracts. These findings are in strong accordance with a recent study in which a plant-derived secondary metabolite has been shown to induce cell cycle assay and consequent apoptosis in HeLa cells^20^.

### Analysis of the apoptotic pathway in HeLa cervical cancer cells

**Figure 5:**
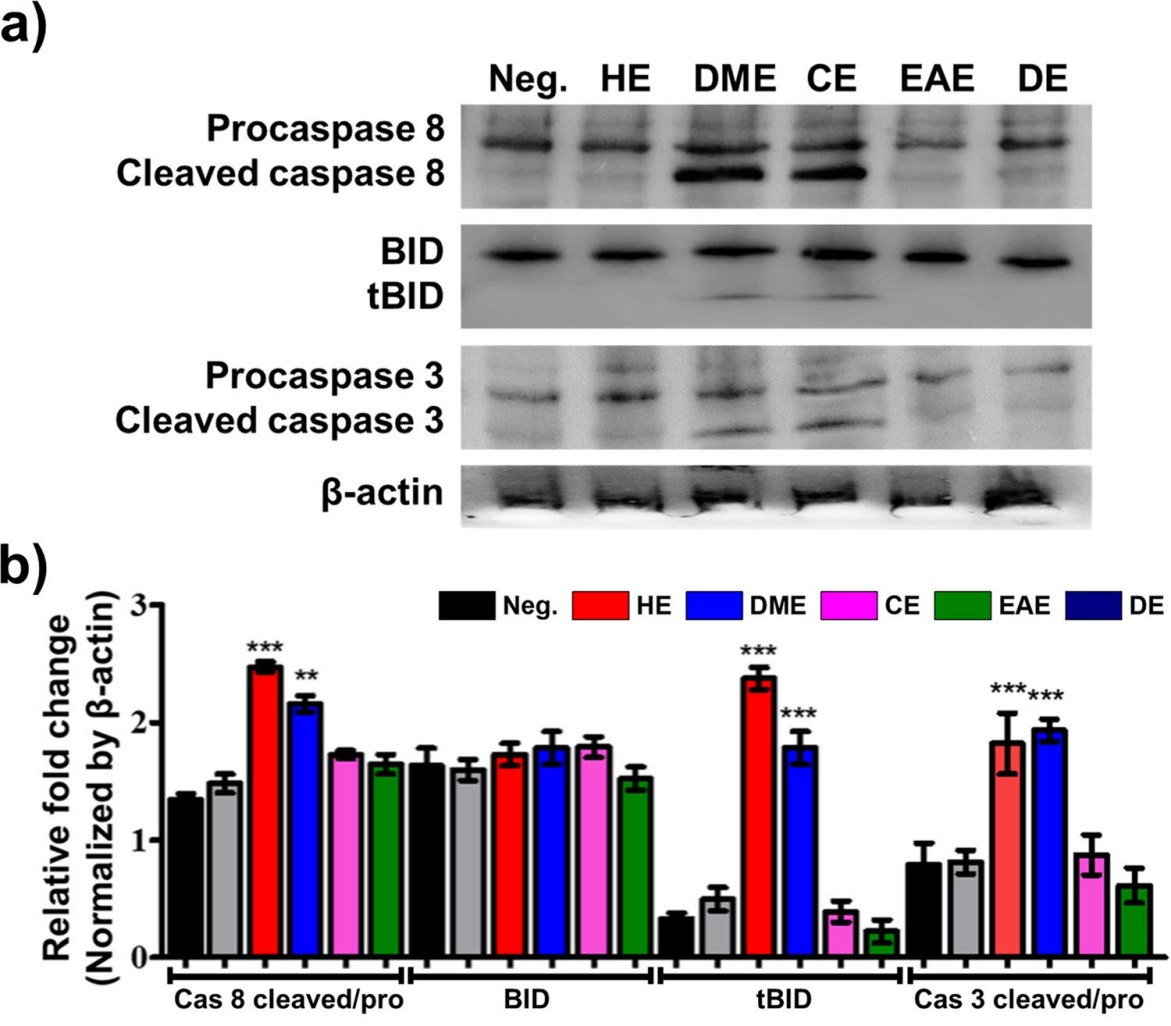
Evaluation of the apoptosis-related protein levels in HeLa cells treated with extracts of *M*. *glyptostroboides* extracts (50 µg/ml). (**a**) Western blot analysis and (**b**) Densitometry quantification of the respective proteins was evaluated by Image J software, and results were normalized with β-actin. **p < 0.01 and ***p < 0.001 indicates statistical significances compared to negative control.

**Figure 6:**
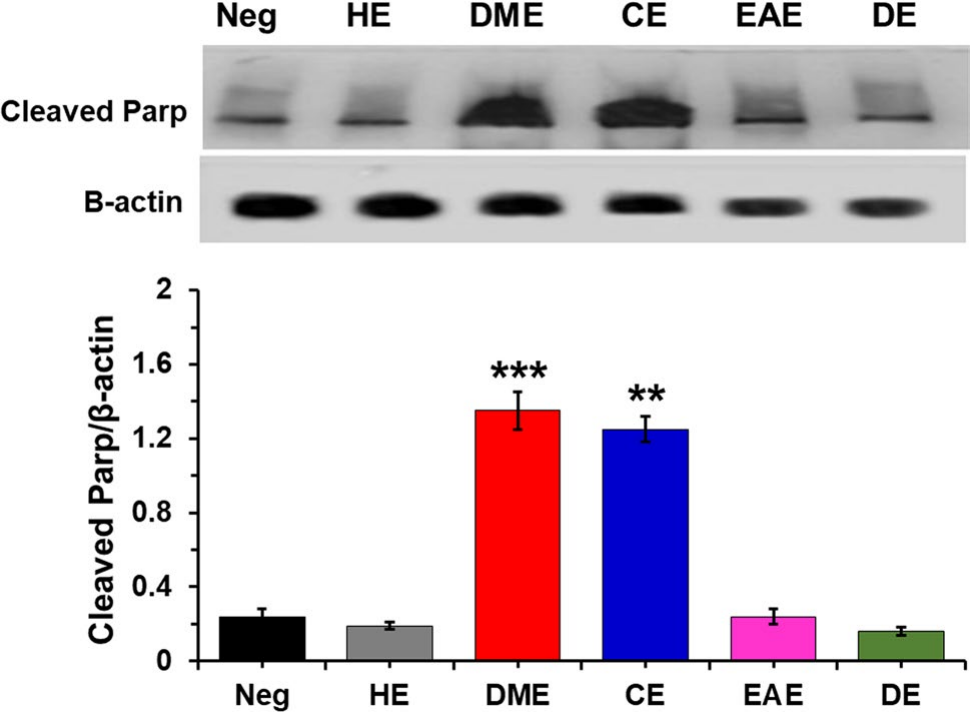
Evaluation of the cleaved Parp level in the HeLa cells treated with extracts of *M. glyptostroboides* extracts (50 µg/ml). **p < 0.01 and ***p < 0.001 indicates statistical significances compared to negative control.

Apoptosis is an important cellular process, and it is divided into two main pathways—extrinsic and intrinsic. To identify the apoptotic pathway induced in HeLa cells by the *M. glyptostroboides* extracts, we performed western blotting to evaluate the changes in the protein levels of the extrinsic apoptosis pathway components BID, Cleaved Caspase (Cl Cas)-3, and Cl Cas-8. Cas-8 is the initiator of the extrinsic apoptosis pathway. Stimulation of the death receptor activates Cas-8, which then cleaves BID^25^. Cleaved BID, also known as truncated BID (tBID), induces the intrinsic pathway. Figure 5b shows the quantification of the data from the western blot analysis of Fig. 5a. The Cl Cas-8 level increased approximately 2.4- and 2.1-folds in HeLa cells treated with the DME and CE extracts, respectively, relative to the level in the negative control. Upregulated Cl Cas-8 levels increased the cleavage of BID into tBID. As shown in Fig. 5b, the tBID levels in HeLa cells treated with the DME and CE extracts were 2.3-, and 1.7-fold more higher, respectively, which were significantly different than that in the negative control. The level of Cl Cas3, which is a member of the cysteine-aspartic acid protease family, was significantly increased in the cells treated with the DME and CE extracts relative to the level in the negative control. These results strongly support that the DME and CE extracts activate caspase-8 to induce apoptosis in HeLa cells via the extrinsic pathway. Also, the significantly higher amount of cleaved Parp, a well-known marker of DNA damage and apoptosis^26^, was noticed in the cells treated with the DME and CE (Fig. 6), suggesting the apoptotic potential of these extracts.

**Figure 7:**
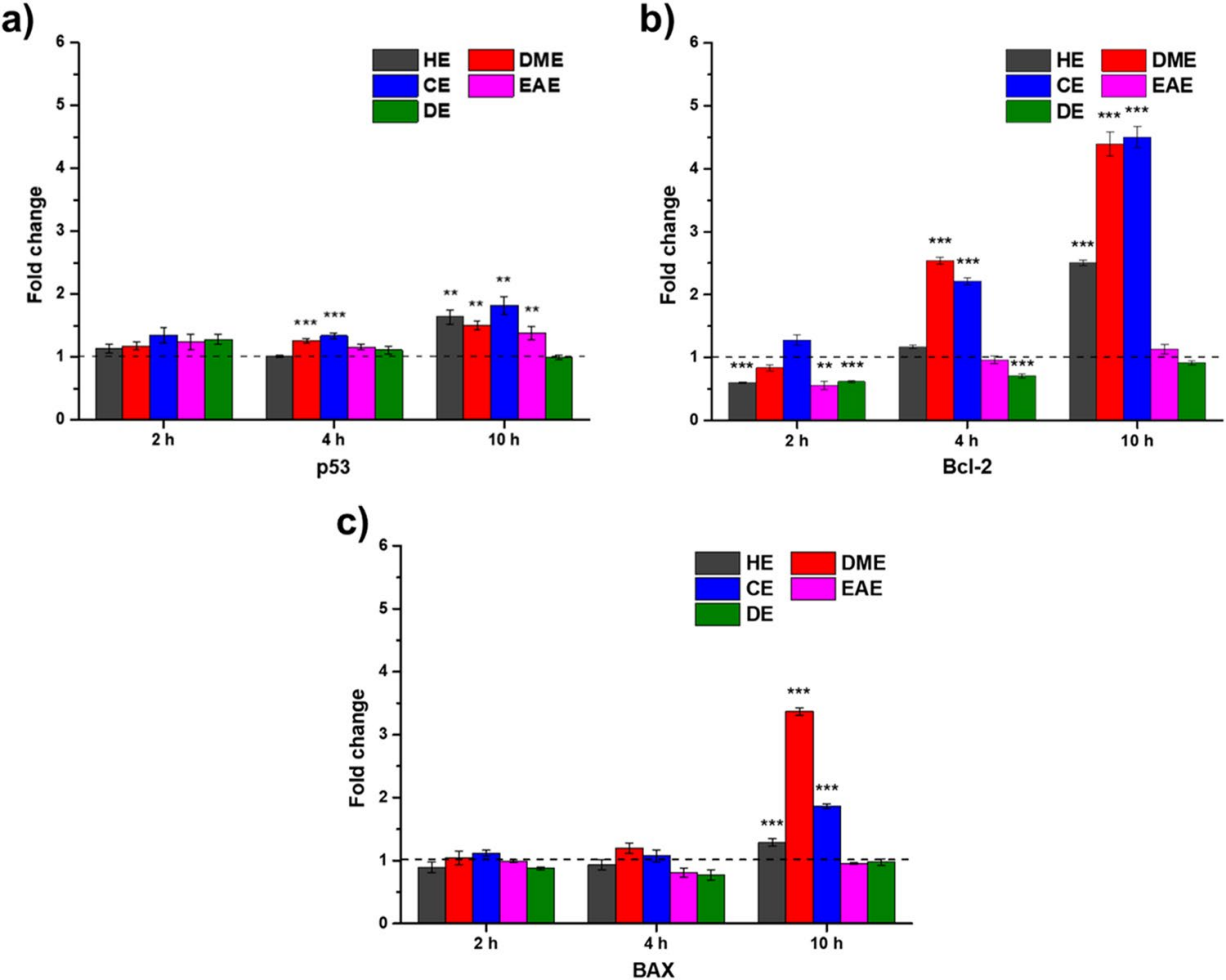
Gene expression analysis in HeLa cells. (**a**) *p53*, (**b**) *Bcl-2*, and (**c**) *BAX* expression. **p < 0.01 and ***p < 0.001 indicates statistical significances compared to negative control.

The production of tBID in the extrinsic pathway may affect the intrinsic pathway. Therefore, we evaluated the mRNA levels of BAX, p53, and Bcl-2, which are involved in the intrinsic pathway. Figure 7 shows the temporal mRNA levels of Bcl-2, BAX, and p53 in HeLa cells as assessed by qRT-PCR. Interestingly, Bcl-2 mRNA level increased with time in cells treated with the HE, DME, or CE extract, and after 10 h, 2.5-, 4.3-, and 4.5-folds increase were observed, respectively, relative to the level in the negative control group. After 10 h of treatment with the HE, CE, or DME extract, BAX mRNA level was upregulated by 1.3-, 1.9-, and 3.4-folds, respectively. Additionally, p53 mRNA level increased approximately 1.5-fold upon 10 h of treatment with the HE, DME, or CE extract (Fig. 7a). However, no significant changes were observed with the EAE and DE extracts. BAX mRNA level was increased by the DME and CE extracts in a time-dependent manner, but Bcl-2 mRNA level also increased (Fig. 7b,c). Both BAX and Bcl-2 are involved in the intrinsic apoptosis pathway. BAX is a pro-apoptotic protein regulated by the tumor suppressor protein p53. Conversely, Bcl-2 is an anti-apoptotic protein that binds to BAX. When HeLa cells were treated with the HE, DME, and CE extracts, the intrinsic pathway was distracted by the upregulated Bcl-2 levels while being induced by the upregulated p53 and BAX levels. In addition, reactive oxygen species, which is one of the major factors in the intrinsic apoptosis pathway, was found to be different at the chemical level, however, it did not cause any difference in the cells (Supplementary Fig. S3).

**Figure 8:**
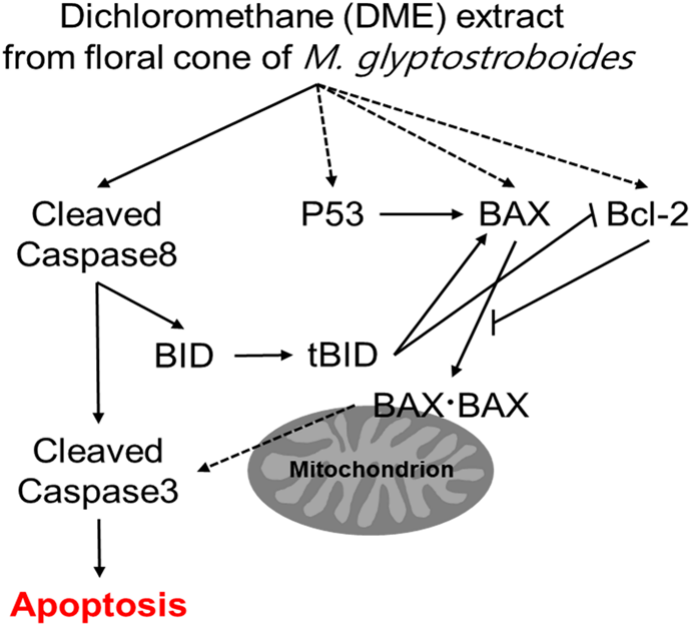
Working model for the mechanism underlying the induction of apoptosis in HeLa cells by the DME extract. The dotted arrows indicate that more evidence is needed to establish a correlation. The solid arrows denote activation. "–" indicates inhibition.

Taken together, *M. glyptostroboides*-derived organic extracts, particularly the DME extract, which may contain anticancer terpenoid compounds^13^ induced Cl Cas8, resulting in the increased cleavage of BID into tBID, which probably promotes the intrinsic pathway. However, Bcl-2, which increases simultaneously with BAX and p53 levels, is thought to interfere with the intrinsic pathway (Fig. 8).

## Conclusions

The DME organic extract derived from the floral cones of *M. glyptostroboides* containing anticancerous quinone, terpenes and steroid components showed high cytotoxicity in cervical cancer cells (HeLa) and low toxicity in normal cells (COS7). Furthermore, Hoechst 33342 staining, cell cycle assay, western blotting, and RT-PCR results showed that the cytotoxicity of the extract resulted from the induction of the extrinsic apoptosis pathway. In particular, cleaved Cas8, which plays an important role in extrinsic apoptosis, was upregulated by 27.17-fold relative to the level in the negative control, indicating the anticancer potential of the DME extract. However, further research is needed to unravel the death receptor and corresponding extrinsic pathway to elucidate the exact apoptosis mechanism induced by the DME extract and the relevant biomedical potential.

## Supplementary Information

Supplementary Information.

## Data Availability

The authors declare that all the data supporting the finding of this study are available within the article and from the corresponding author on reasonable request.
