# Supplementary material for: Metasequoia glyptostroboides potentiates anticancer effect against cervical cancer via intrinsic apoptosis pathway
Source: Sci Rep. 2021 Jan 13;11:894. doi: 10.1038/s41598-020-79573-8 (PMC7806586; doi:10.1038/s41598-020-79573-8)
Supplement: Supplementary file 1 — Supplementary Information. [file 41598_2020_79573_MOESM1_ESM.docx]

**Supplementary Information**

*Metasequoia* *glyptostroboides* potentiates anticancer effect against cervical cancer via intrinsic apoptosis pathway

Hoomin Lee^1,$^, Cheolwoo Oh^1,$^, Suji Kim^1^, Debasish Kumar Dey^2^, Hyung Kyo Kim^3^, Vivek K. Bajpai^4,^*, Young-Kyu Han^4,^* & Yun Suk Huh^1,^*

^1^Department of Biological Engineering, Biohybrid System Research Center (BSRC), Inha University, Incheon 22212, Republic of Korea

^2^Department of Biotechnology, Daegu University, Gyeongsan, 38453, Republic of Korea

^3^Department of Biomaterials Research Center, GENPEAU Corporation, Incheon 21990, Republic of Korea

^4^Department of Energy and Materials Engineering, Dongguk University-Seoul, 30 Pildong-ro 1-gil, Seoul 04620, Republic of Korea

**^$^Equally contributed**

**Running head:** Anticancer potential of *M. glyptostroboides*

***** Authors to whom correspondence should be addressed: vbiotech04@gmail.com (V.K. Bajpai); E-mail: ykenergy@dongguk.edu (Y.-K. Han); E-mail: yunsuk.huh@inha.ac.kr (Y.S. Huh)

**Table S1.** Primer sequences used in RT-qPCR.

| **Gene name** |  | **Sequences** |
| --- | --- | --- |
| ***Bcl-2*** | Sense | 5'-ATC GCC CTG TGG ATG ACT GA-3' |
|  | Antisense | 5'-GGG CCG TAC AGT TCC ACA A-3' |
| ***BAX*** | Sense | 5'-AGC GAC TGA TGT CCC TGT CT-3' |
|  | Antisense | 5'-CTC CCG CCA CAA AGA TGG TC-3' |
| ***P53*** | Sense | 5'-GGC CCA CTT CAC CGT ACT AA-3' |
|  | Antisense | 5'-TGA GGT AGG TGC AAA TGC CA-3' |
| ***β-actin*** | Sense | 5'-CCG AGG ACT TTG ATT GCA CAT TG-3' |
|  | Antisense | 5'-TGG GGT GGC TTT TAG GAT GG-3' |

**Table S2.** Chemical component analysis of DME using GC-MS.

| No. | Retention Time (RT) | Compound | Area (%) | Method |
| --- | --- | --- | --- | --- |
| 1 | 7.13 | 1,3,6-Heptatriene | 0.04 | GC-MS |
| 2 | 15.21 | Propionamide | 0.05 | GC-MS |
| 3 | 20.84 | Ethanedicarboxamide | 0.04 | GC-MS |
| 4 | 22.51 | 2,4,6-Octatriene | 0.46 | GC-MS |
| 5 | 36.54 | Ethanol | 0.1 | GC-MS |
| 6 | 37.35 | Isoaromadendrene epoxide | 0.29 | GC-MS |
| 7 | 42.11 | Phenylethanolamine | 0.07 | GC-MS |
| 8 | 44.66 | Butyramide | 0.12 | GC-MS |
| 9 | 46.72 | Hexadecanoic acid | 0.41 | GC-MS |
| 10 | 51.6 | Benzeneethanamine | 0.08 | GC-MS |
| 11 | 51.89 | Methyl 9,10-octadecadienoate | 0.18 | GC-MS |
| 12 | 52.09 | 9-Octadecenoic acid | 0.31 | GC-MS |
| 13 | 52.91 | N-Neopentylpropanamide | 0.06 | GC-MS |
| 14 | 55.65 | Quinoline | 0.09 | GC-MS |
| 15 | 56.67 | 4-Methoxyamphetamine | 0.15 | GC-MS |
| 16 | 57.00 | 2,4-Dimethylamphetamine | 0.07 | GC-MS |
| 17 | 57.65 | 2,5(1H,3H)-Quinolinedione | 0.25 | GC-MS |
| 18 | 58.20 | Phosphine oxide | 11.97 | GC-MS |
| 19 | 58.36 | Ferruginol | 14.58 | GC-MS |
| 20 | 59.59 | 4H-Pyrrolo[3,2,1-ij]quinoline | 0.84 | GC-MS |
| 21 | 60.03 | 3,4'-Isopropylidenediphenol | 0.23 | GC-MS |
| 22 | 60.86 | 1,4-dimethyl-2-quinolinethione | 0.19 | GC-MS |
| 23 | 61.38 | Taxodione | 2.21 | GC-MS |
| 24 | 61.75 | 3-Bromo-4,5-dihydroxybenzaldehyde | 0.47 | GC-MS |
| 25 | 61.84 | Estradiol | 0.47 | GC-MS |
| 26 | 62.13 | 5H-Indeno[1,2-b]pyridine | 0.28 | GC-MS |
| 27 | 63.56 | acridine, Chrysene | 0.42 | GC-MS |
| 28 | 63.70 | 2-methoxy-4-methyl-N-phenlyanilin | 0.2 | GC-MS |
| 29 | 63.96 | Benzenamide | 0.44 | GC-MS |
| 30 | 64.4 | 2-Adamantanone semicarbazone | 0.18 | GC-MS |
| 31 | 64.54 | 4-Amino-2-oxy-furazan-3-carboxylic acid | 0.23 | GC-MS |
| 32 | 64.86 | 3-Isoxazolecarboxylic acid | 4.53 | GC-MS |
| 33 | 65.19 | 3,4,5-Tribromopyridine | 0.18 | GC-MS |
| 34 | 65.99 | Phenanthrenone | 10.8 | GC-MS |

**Table S2.** Continued on

| 35 | 66.14 | (+)-Isosarcophytoxide | 0.59 | GC-MS |
| --- | --- | --- | --- | --- |
| 36 | 66.34 | N-(3-Aminopropyl)methanesulfoamid | 0.34 | GC-MS |
| 37 | 67.34 | dibenzopyrone | 2.29 | GC-MS |
| 38 | 67.56 | Mrophinan | 0.77 | GC-MS |
| 39 | 67.85 | n-Octanoic acid | 0.77 | GC-MS |
| 40 | 68.88 | 2-Phenanthrenol | 3.76 | GC-MS |
| 41 | 69.67 | Aniline | 0.13 | GC-MS |
| 42 | 69.77 | 9-Octadecenamide | 0.31 | GC-MS |
| 43 | 70.54 | 13,14-Deoxycoleon | 38.32 | GC-MS |
| 44 | 76.96 | 1-Naphthalenesulfonic acid | 0.24 | GC-MS |
| 45 | 79.12 | Heptasiloxane | 0.31 | GC-MS |

#
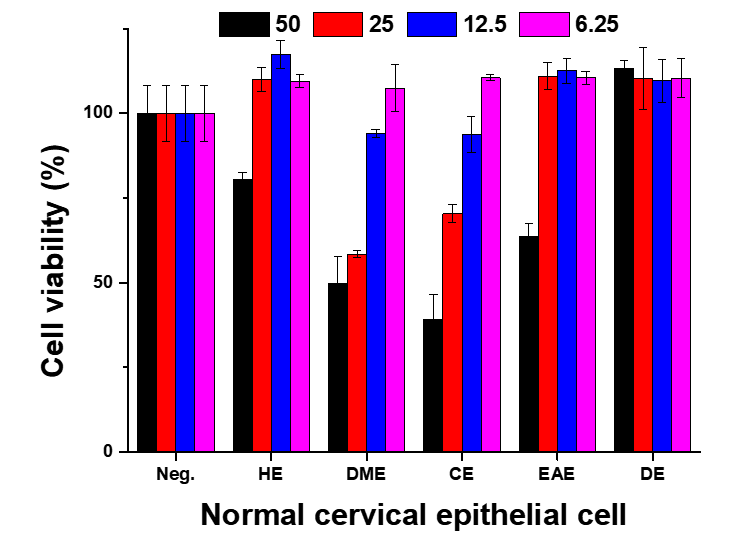


# Figure S1. Anticancer effects of various extracts of *M. glyptostroboides* on the normal cervical epithelial cell.

#
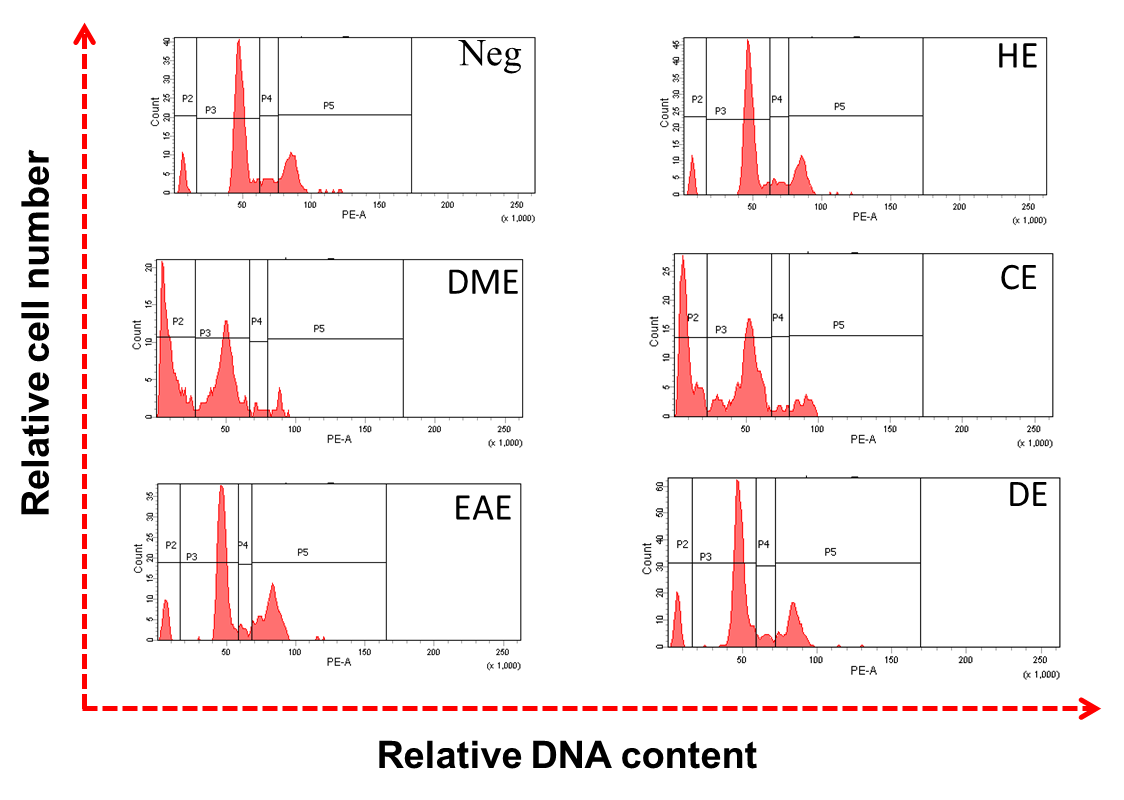


# Figure S2. FACS analysis of HeLa cells were treated with *M. glyptostroboides* extracts of 50 µg/mL for 24 h.

#
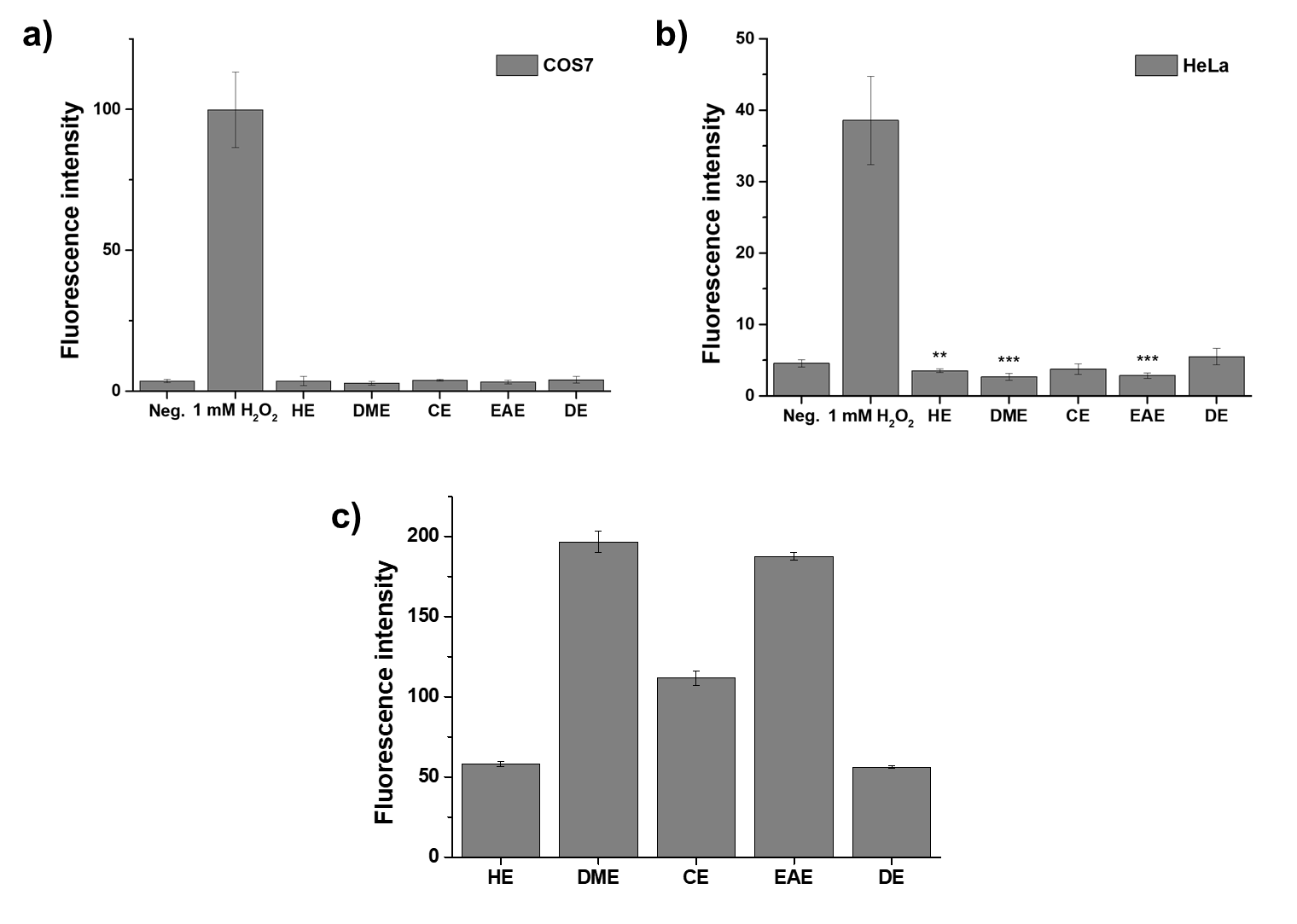


# Figure S3. DCF-DA assay at the cellular and chemical levels. *In vitro* DCF-DA assay on a) COS7 and b) HeLa cells were incubated with *M. glyptostroboides* extracts of 50 µg/mL for 6 h, c) chemical level. **p < 0.01 and ***p < 0.001 indicate statistical significances compared to negative control.

#
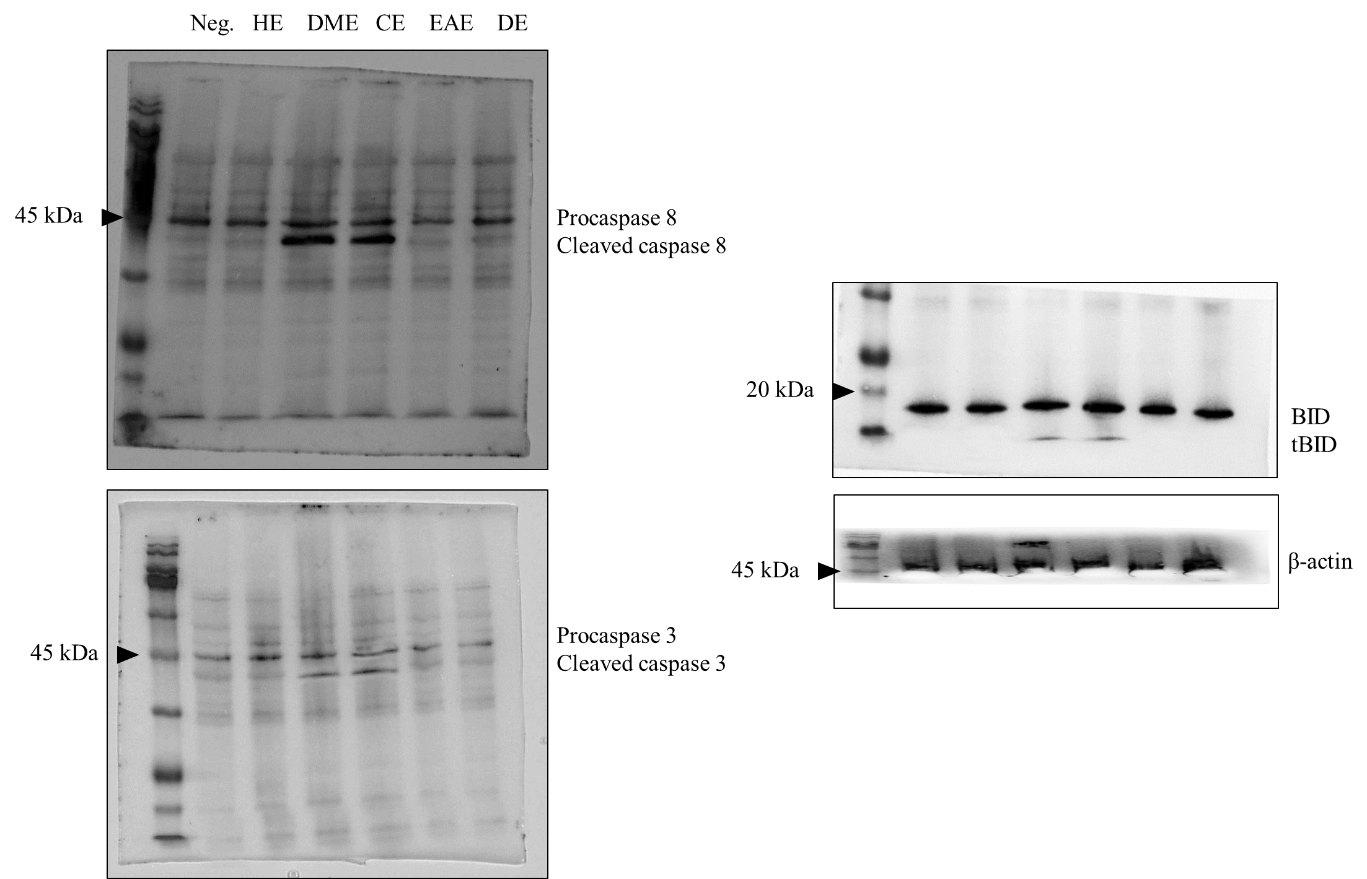


**Figure S4.** The whole images representing caspase-8 and cleaved caspase-8, caspase-3, and cleaved caspase-3 with molecular weight markers.
